# Research Progress of Wood-Based Panels Made of Thermoplastics as Wood Adhesives

**DOI:** 10.3390/polym14010098

**Published:** 2021-12-28

**Authors:** Xianfeng Mo, Xinhao Zhang, Lu Fang, Yu Zhang

**Affiliations:** 1 Co-Innovation Center of Efficient Processing and Utilization of Forest Resources, Nanjing Forestry University, Nanjing 210037, China; moxianfeng47@hotmail.com; 2 College of Furnishings and Industrial Design, Nanjing Forestry University, Nanjing 210037, China; zhangxinhaonjfu@163.com (X.Z.); ZY19980427@163.com (Y.Z.)

**Keywords:** thermoplastic resin, formaldehyde-free adhesive, hot-pressing technique, wood-based panels

## Abstract

When thermoplastic resins such as polyethylene (PE) and polypropylene (PP) are selected as wood adhesives to bond wood particles (fibers, chips, veneers) by using the hot-pressing technique, the formaldehyde emission issue that has long existed in the wood-based panel industry can be effectively solved. In this study, in general, thermoplastic-bonded wood-based panels presented relatively higher mechanical properties and better water resistance and machinability than the conventional urea–formaldehyde resin-bonded wood-based panels. However, the bonding structure of the wood and thermoplastic materials was unstable at high temperatures. Compared with the wood–plastic composites manufactured by the extruding or injection molding methods, thermoplastic-bonded wood-based panels have the advantages of larger size, a wider raw material range and higher production efficiency. The processing technology, bonding mechanism and the performance of thermoplastic-bonded wood-based panels are comprehensively summarized and reviewed in this paper. Meanwhile, the existing problems of this new kind of panel and their future development trends are also highlighted, which can provide the wood industry with foundations and guidelines for using thermoplastics as environmentally friendly adhesives and effectively solving indoor pollution problems.

## 1. Introduction

The development of the wood-based panel industry has played a prominent role in alleviating the contradiction between the supply and demand of wood products, protecting forest resources and the ecological environment [1,2,3]. Plywood, fiberboard and particleboard are the three predominant types of wood-based panels and have been widely developed as alternative materials for furniture manufacturing, decorative panel production, wood structure building boards and other fields because of their stable physical properties, wide sources of raw materials and good end-use properties [4]. However, wood adhesives, the essential components predominantly used in manufacturing wood composites, are mainly formaldehyde-based materials, such as urea–formaldehyde (UF) resin, phenol–formaldehyde (PF) resin and melamine urea–formaldehyde (MUF) resin [5,6]. These adhesives have the advantages of good adhesion and technological maturity, but they release formaldehyde when producing and using the wood composites [7]. In 2004, the International Agency for Research on Cancer reclassified formaldehyde from “probably carcinogenic to humans” to “carcinogenic to humans”. The European Union, the USA, China and Japan now have legislation regulating the allowed levels of formaldehyde emission from wood-based products [8]. These events culminated with the implementation of the most stringent formaldehyde release standard in 2017. The new allowable emission levels vary by product, but the limits (not to be exceeded) are in the range of 50 to 100 ppb [9], which put forward much higher requirements regarding wood adhesives. Significant efforts have been dedicated to reducing synthetic (anthropogenic) CH_2_O emission from the resins, principally by reducing the F/U mole ratio and modifying the formulation [10,11] and adding CH_2_O scavengers [12]. However, these cannot solve the problem from the root cause. Alternative “no-added-formaldehyde” (NAF) resins, such as those based upon soybean, starch and lignin, have also captured great attention [13,14]. However, they only occupy minor positions in this vast market due to their poor water resistance, mobility and stability [15,16,17].

In recent years, the use of thermoplastic resins to bond wood materials has become a promising method to manufacture wood-based panels due to its advantages of environmental friendliness, good flexibility, water resistance and processability [18,19,20,21,22]. The study of thermoplastics as wood adhesives began in the 1990s and was first proposed by Dr. Han of Kyoto University [23]. He indicated that PP and modified PP have the merit of unique adhesion performance when combined with wood components. The study of the use of various thermoplastics as wood adhesives in China began in the mid-1990s and was proposed by Wang from the Chinese Academy of Forestry [24]. In 2005, the research results of thermoplastic-bonded plywood obtained the authorization of China’s national invention patent, and it has completed independent intellectual property rights. In 2006, it was awarded as China’s national key new product. The preparation methods and performance of the thermoplastic-bonded wood-based panels have been continuously studied and developed in recent years [25,26,27,28,29,30,31,32,33,34,35]. This paper aims to summarize the recent progress in thermoplastic-bonded wood-based composites, focusing on their processing technology, bonding mechanism and physical–mechanical performance. Meanwhile, the existing problems and the future development trends of thermoplastics as wood adhesives are highlighted.

## 2. Processing Technology of Thermoplastic-Bonded Wood-Based Panels

### 2.1. Raw Materials

Under certain pressure, melted thermoplastics have the merit of unique adhesion performance when combined with wood or other plant components. Both virgin and recycled thermoplastics can be used for compounding with wood, but the melting temperature of the selected thermoplastics must be lower than the degradation temperature of wood (200 °C). Thermoplastic resins that have been studied included polypropylene (PP), polyethylene (PE), polyvinyl chloride (PVC), polystyrene (PS) [35] and poly-β-hydroxybutyrate (PHB) [36]. Thermoplastics are solvent-free and have various forms, such as granules, powders, sheets, films, wires and blocks. Various forms of wood, such as wood veneer, wood particle and wood fiber, can be directly bonded with thermoplastics. In most of the existing studies, thermoplastic films were preferentially selected to bond wood veneers to prepare wood–plastic plywood [29,33,34,35]. When bonding wood particles, both thermoplastic films and thermoplastic powders have been popular choices [27,31,32].

### 2.2. Processing Technology of Wood–Plastic Plywood

Similar to the processing methods of commercially available medium-density fiberboard (MDF), particleboard or plywood, thermoplastic-bonded wood-based panels can be manufactured by the traditional hot-pressing technique [24]. There is no gluing stage in the whole production process, but the panel must be cold-pressed immediately after it is taken out of the hot press [35,36,37,38].

**Figure 1:**
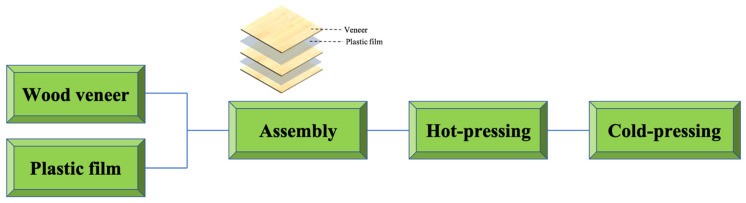
The scheme of wood–plastic plywood fabrication.

**Figure 2:**
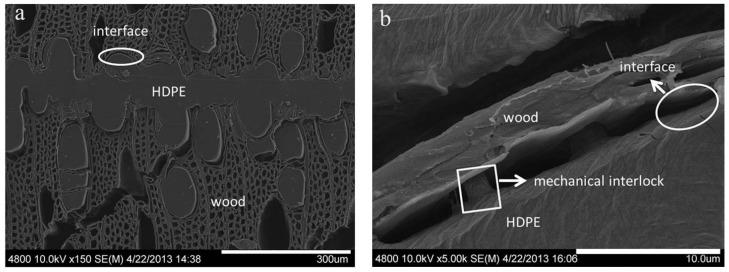
Derived from Ref [26]. Bonding interface between HDPE film and wood veneer: (**a**) magnification ×150; (**b**) magnification ×5000.

The manufacturing process of wood–plastic plywood is relatively simple, as shown in Figure 1. The setup requires only plastic films between every two wood veneers, and the material is then fabricated using combined hot pressing and cold pressing. During the preparation of wood–plastic plywood, the thermoplastic film is heated and melted into the porous structure of the wood veneer, and then cooled and solidified to form the “glue nail” microstructure interface phase (Figure 2). In the heating stage, thermoplastics experience a transition from liquid to solid, playing the role of wood adhesive. Both the hot-pressing conditions and wood surface properties affect the bonding strength. Due to the characteristics of the thermoplastic film, shrinkage inevitably occurs during the formation of the wood–plastic interface. The shrinkage residual stress must be compensated in the cold-pressing stage; otherwise, the interfacial debonding failure of composites can easily occur under an external load [39,40].

### 2.3. Processing Technology of Wood–Plastic Particleboard

**Figure 3:**
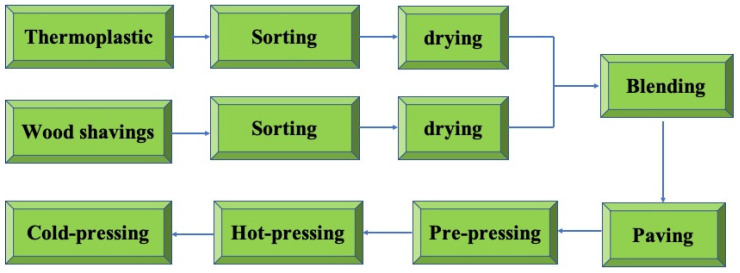
The scheme of wood–plastic particleboard fabrication.

The combination of wood flour and thermoplastics by extrusion or injection methods has become a standard practice. In wood–plastic composites (WPCs) manufactured by these two technologies, thermoplastics serve as a continuous phase, while wood flour is a dispersed phase to provide strength and stiffness [41]. WPCs can be made into solid products with various shapes, sizes and colors, and their products have been widely used in industry [42]. However, the use of the extrusion process is quite limited for large-sized particles due to its high machinery costs [43], and multi-dimensional products can only be prepared by injection molding [44,45]. To overcome these problems, the concept of bonding wood particles with the use of thermoplastics by the hot-pressing technique has been developed (as shown in Figure 3) [46,47,48,49,50]. Panels prepared by this method are called wood–plastic particleboard. On the one hand, it has the advantages of higher productivity and a lower pressing pressure requirement. The proportion of wood components can reach up to 80% by volume using this process, and the natural wood structure can be maintained. On the other hand, this method can obtain large-sized boards with a length of 2440 mm, a width of 1220 mm and a thickness of 3 to 40 mm [51].

For wood–plastic particleboard, the matrix can be ordinary wood shavings or other shavings prepared from agricultural residues such as bamboo, straw or cotton stalk [52]. Due to the wide range of raw materials, obtaining a very uniform blend of shavings and thermoplastic mixture has become one of the key points in the manufacturing technology of wood–plastic particleboard [53,54]. It is very important to find the proper moisture content, particle size and particle shape of the raw materials. A general rule for mixing is that large pieces of wood shavings should be matched with large pieces of thermoplastic, and small pieces of shavings matched with small pieces of thermoplastic. Increasing the fiber aspect ratio can produce a larger contact area between the fiber and plastic matrix, which is able to improve the composites’ performance [55,56]. Qi [31,57] prepared cotton stalk–HDPE-oriented composites. It was found that the mechanical properties of composites prepared with long cotton stalk bundles were 2 to 3 times higher than those prepared with short or granular cotton stalk bundles. In the meantime, it is also necessary to focus on the processing cost when recycled plastic is utilized. For commonly used recycled plastics, such as PE and PP, directly sorting, cleaning and pulverizing before use is an ideal method.

Since no adhesive with initial tack is added in the manufacturing process of wood–plastic particleboard, and wood particles and thermoplastics are only physically mixed, their combinations are very scattered. Therefore, ensuring that the is mixture formed into a uniform mat and effectively transported during the assembly process has become another challenge for wood–plastic particleboard manufacturing [58]. Li et al. [59] prepared wood-fiber-reinforced PP composites by flat hot-pressing and compression molding. Results showed that when the wood fiber content reached 80%, the composites displayed a good flexural modulus and impact strength for both of the methods. Compared with the flat hot-pressing method, composites produced by the compression molding method had higher density and a better flexural modulus. Meanwhile, the composites manufactured by the flat hot-pressing method showed better surface wettability and impact strength. It is also worth noting that efficient bonding of the wood–thermoplastic system can be achieved only when the thermoplastic binder is sufficiently plasticized to be easily extruded into the pores of the wood shavings. Therefore, a low-pressure preheating step is necessary for the production of wood–plastic particleboard [60]. In order to utilize agricultural and forestry wastes, He et al. [52] prepared PP wood–plastic composites filled with rice straw powder, rice husk powder, wood powder and bamboo powder, as well as their mixture, using mixed compression molding and layer compression molding, respectively. For the mixed compression molding method, PP was first blended with the powders and then molded. For the layered compression molding, the powders were directly layered on the PP film. The results showed that the mechanical properties, water absorption and moisture absorption performance of the PP composites prepared by mixed compression molding were better than those of layered molding PP composites. The filler can be well-distributed in the melted PP matrix when using the mixed compression molding. By contrast, the wood-based panels prepared by the layered compression molding have a poor interface between the thermoplastic and the fillers.

## 3. Performance of Thermoplastic-Bonded Wood-Based Panels

Fabrication of thermoplastic-bonded wood-based panels with the hot-pressing methods in the laboratory resembles the standard procedure of composite production in industry. Its products are closely comparable to commercial plywood, MDF and particleboard. Compared with natural-based adhesive-bonded wood-based panels, this can offer improved water resistance and relatively simple processing technology.

### 3.1. Environmental Performance

Thermoplastics play the role of wood adhesives in the manufacturing process of thermoplastic-bonded wood-based composites. Since the traditional formaldehyde adhesive has been completely replaced, the most remarkable feature of thermoplastic-glued wood-based panels is their environmental friendliness. Table 1 presents the free formaldehyde content of wood–plastic particleboard as determined by two different test methods [58]. The values measured by these two methods are far lower than that in the “formaldehyde release limit of wood-based decorative materials and their products” standard. It is worth noting that the presence of a small amount of formaldehyde arises from the wood itself, and is called biogenic formaldehyde [61]. All organic wood components contribute to biological formaldehyde’s generation and emission. A great deal of evidence has shown that this kind of formaldehyde mainly arises from lignin. At the same time, the total volatile organic compound (TVOC) release rate of thermoplastic resin wood-based panels was found to be only 0.01 mg/(m^2^·h), which is also far below the value specified in the relevant standards [62,63].

### 3.2. Physical and Mechanical Properties

#### 3.2.1. Water Resistance

Thermoplastic resins, especially polyolefin plastics, are usually hydrophobic materials. They do not absorb water, which gives thermoplastic-bonded wood-based composites excellent water resistance. In the bonding process, thermoplastics can not only be filled in the porous structure of wood, but also cover part of the hygroscopic wood surface. The speed of water entering the wood is therefore slowed down, and the moisture penetrating into the wood is decreased accordingly [25,36,50,64]. Wang et al. [58] tested the water resistance of several particleboards. It was found that both the 2 h and 24 h thickness swelling (TS) of the thermoplastic-bonded particleboards were lower than 3%. After soaking in boiling water for 2 h, the internal bonding strength (IBS) of thermoplastic-bonded particleboards was higher than the traditional sliced veneer-decorated wood-based panels. Fang et al. [25] found that the water resistance of plywood bonded with PE film was much better than that of urea–formaldehyde (UF) resin-bonded panels. After soaking for 168 h, the water absorption and thickness expansion of PE film plywood were 85.8% and 7.7%, respectively, which are 18.8% and 4.9% lower than those of UF resin plywood. Due to their low expansion rate and good dimensional stability in a humid environment, thermoplastic-bonded wood composites will have extensive application prospects in cement formwork and outdoor packaging. As shown in Table 2, the larger the amount of thermoplastic resin used, the better the water resistance of the wood-based panel. However, it will have an adverse impact on the mechanical properties of the panel if the usage is excessive. For wood–plastic particleboards, most authors indicated that the panel achieves optimal resistance to bending forces when the content of wood particles is in the range of 40% to 60%. For wood–plastic plywood, when the film thickness is greater than 0.1 mm (adhesive dosage is around 100 g/m^2^ in double glue line), it can meet the strength requirement for interior products (type II).

#### 3.2.2. Mechanical Properties

Thermoplastics have the merit of unique adhesion performance when composited with wood components [66,67,68,69]. Since there is no chemical reaction between thermoplastics and wood, mechanical interlock is the main bonding mechanism of thermoplastic-bonded wood composites. When a specific thermoplastic is selected as the wood adhesive, the appropriate hot-pressing temperature must be determined [22,25,35,70]. The hot-pressing temperature affects the thermoplastic viscosity. It is believed that the lower the viscosity, the deeper the penetration depth [71]. Generally, the hot-press temperature should be 15 to 35 °C higher than the melting temperature of the thermoplastic so that it can fully flow into and between the wood cell lumens and voids, helping to form more glue nails. Luedtke et al. [72] showed that poly(lactic acid) (PLA) bonded plywood prepared at >160 °C had higher tensile strength than those formed at 140 °C, which was associated with a thinner bondline and greater PLA migration away from the bondline, consistent with lower melt viscosity under high temperatures. The deeper PLA penetration depth contributed to the better physical interlocking of the PLA within the wood ultrastructure. However, it should be noted that the thickness of the adhesive bondline also has a great impact on the quality and performance of the adhesive. Over-penetration may occur if the hot-pressing temperature is too high, which will deteriorate the strength of thermoplastic-bonded wood composites [38,70]. Due to the high viscosity of thermoplastics, they usually take a long time to reach the fully molten state, and then gradually spread out on the veneer surface to complete the penetration step. Fang et al. [37] prepared formaldehyde-free plywood using PE film as a wood adhesive and found that the hot-pressing time and hot-pressing temperature have an interactive effect on the plywood tensile shear strength.

The properties of thermoplastic-bonded wood-based panels are closely related to the thermoplastic type and their dosage, as shown in Table 3 [25,36,64,66,73]. PE is one of the most widely used thermoplastic polymers and has a very simple structure. It has been widely studied in the wood industry recently because of its low price and good water resistance [18,25,29,30,57,70,74]. Results showed that the stiffness of thermoplastic PE is lower than that of thermosetting UF resin, but its plasticity can give PE film-bonded plywood stronger bending failure resistance and therefore a higher MOR value. The bonding strength and elastic modulus the PE film-bonded poplar plywood were found to be similar to those of UF resin-bonded poplar plywood (the difference was less than 1%). In addition, due to the high-temperature softening property of PE and its poor compatibility with wood, the prepared PE plywood can only meet the strength requirements of indoor materials (type II). When the outdoor material test standard was performed (three-cycle treatment: soaking in boiling water for 4 h, then drying at 63 °C for 20 h and soaking in boiling water for 4 h again), the PE film was completely separated from the wood. PP has better high-temperature resistance compared with PE, but higher energy consumption is required for preparing the panels. Li et al. [75] fabricated eucalyptus plywood using PP film as an adhesive. The tensile shear strength was 1.4 MPa after outdoor aging treatment (type I, the specified value of the standard is 0.7 MPa). Xia [32,76] prepared oriented cotton stalk–PP boards and oriented cotton stalk–HDPE boards under the same processing conditions (hot-press temperature 185 °C, hot-press time 15 min, board density 0.7g/cm^3^). Results showed that the mechanical properties and water resistance of the two directional composite boards were better than those of conventional MDF and particleboard. The 24 h TS value of both boards was less than 3%. When the amount of thermoplastic resin was fixed at 15%, the static flexural strength, elastic modulus and internal bonding strength of the oriented cotton stalk–PP boards were 60.60, 5074.4 and 1.48 MPa, respectively, which are 33%, 13% and 13% higher than those of PE composites. When the mass fraction of thermoplastic resin exceeded 15%, the mechanical properties of the two composites decreased, and the water resistance was further improved. Polyvinyl chloride (PVC) film has the characteristics of lower price and wider availability compared with PP and PE [33,77,78]. It has also been successfully applied to the manufacturing of plywood.

## 4. Technical Problems of Wood-Based Panel Made of Thermoplastic Resin Adhesive

The physical–mechanical properties of this novel formaldehyde-free wood-based panel can meet the strength requirements for different applications by controlling the process conditions. Their technological and environmental benefits confirm their suitability to gradually replace the traditional plywood, particleboard or fiberboard. However, thermoplastics have the characteristics of melting and softening at high temperatures. When the temperature approaches or exceeds the melting point of the thermoplastic, the bonding structure between the thermoplastic and wood cannot be maintained, and the strength of the panel will be lost [25,79]. When thermoplastics are used as adhesives, their incompatibility with wood can present another major problem [18,26,29,36,47]. As shown in Figure 2b, there was a large gap at the interface. Therefore, enhancing the compatibility between thermoplastic and wood raw materials and improving the high-temperature resistance will be the most important work in the future. Multiple review studies related to the improvement of the wood fiber–plastic interfacial interaction have been published [41,80,81,82]. The most popular and most widely described methods are thermal treatment, plasma treatment, alkali treatment, silanization, acetylation, maleation, acrylation or isocyanate treatment. For wood–plastic particleboard, various modification methods that have been successfully applied in WPCs are essentially applicable. Meanwhile, for a wood veneer–plastic film bonding system, the interfacial methods are relatively limited due to the size limitation. Here, we simply focus on some effective methods for wood–plastic plywood.

Giving the wood veneer or plastic film new chemical properties has been proven to be the most effective solution [30,83,84]. Zhou et al. [29,85] grafted oxygen- and nitrogen-containing functional groups onto the surfaces of plastic bags using an industrial atmospheric dielectric-barrier discharge plasma system. Due to the enhanced surface polarity and wettability, formaldehyde-free plywood with a high bonding strength (0.82 MPa) was successfully fabricated. Fang et al. [26] introduced the bifunctional structure of vinyltrimethoxysilane hydrolysate into a wood veneer and successfully established a chemical bridge between the wood veneer and PE film. The bonding interface gap decreased to a single molecular scale because of the silane surface treatment, resulting in an increase in tensile shear strength by 293.2%. The temperature point at which the panel strength sharply decreased raised from 140 to 180 °C, indicating that the interfacial improvement was also of benefit to the thermal stability of the composites.

Physical modification has also attracted extensive attention because of its unique advantages. In order to promote the penetration of thermoplastics in wood, some scholars have used a self-made hole rolling machine to roll dense blind holes on the surface of the wood veneer [86]. This method helped to form a more stable “dendritic glue nail” microstructure and contributed to improving the bonding strength and dimensional stability of the PE film-bonded wood-based panel. The perforation of the thermoplastic film was also conducive to increasing the mechanical interlocking between the plastic and wood [87]. This is because the heat transfer rate was accelerated during the hot pressing. However, the efficiency of these methods is low, and the self-strength of wood veneer or plastic films can easily be reduced. Thermal treatment will not only degrade hemicellulose in wood, but also lead to the migration or volatilization of various extractives to the wood surface [88]. Therefore, an alternative modification method has been explored as a strategy to improve the compatibility of the wood veneer and hydrophobic thermoplastic without contaminating the environment. However, the high temperature usually reduced the elastic modulus of wood. The key technology of thermal treatment was to select appropriate parameters, such as treating temperature and treating medium, so as to effectively change the surface properties of wood without reducing its mechanical properties. To date, there have been several reports of improved dimensional stability, water resistance and biological resistance [89,90,91], but little improvement in flexural properties has been reported. Future work should focus on exploring environmentally friendly modification methods, with low energy consumption and low cost, that are conducive to industrial production. At the same time, the mechanism of various modification methods should be further clarified to add new content to the development of high-performance green wood adhesives.

## 5. Conclusions

Thermoplastic-bonded wood-based panels prepared using the hot-pressing technique have the characteristics of no formaldehyde emission and excellent water resistance. This has become an ideal alternative material for furniture, flooring decoration and so on. To date, a large number of systematic studies have been carried out on these novel panels, but there are still some problems that require further attention.

(1) As for the types of thermoplastics, virgin and single plastics are preferred, among which PP and PE are the most studied. The hot-pressing temperature for preparing the composites is relatively high (above 140 °C). In order to alleviate the defects caused by plastic shrinkage, cooling forming equipment needs to be added for the preparation of thermoplastic-bonded wood-based panels. In general, the manufacturing cost of thermoplastic-bonded wood-based panels is higher than that of traditional trialdehyde-bonded panels.
(2) The incompatibility between thermoplastic and wood has an adverse impact on the mechanical properties. Therefore, thermoplastic-bonded wood-based panels can only find use in some nonstructural applications. To date, numerous modification methods have been tested and proven to counter the incompatibility between hydrophilic wood and hydrophobic thermoplastics, but some of their enhancement mechanisms need to be further clarified.
(3) For thermoplastic-bonded particleboard or fiberboard, obtaining uniformly mixed materials at the lowest cost, ensuring the uniform paving and molding of mixtures and selecting the cold-pressing parameters according to different manufacturing processes still lack data and theoretical support.
(4) The performance evaluation of wood-based panels made with thermoplastic resin adhesives mainly focuses on indexes such as bonding strength, bending resistance and water resistance, while there is a lack of research on the flame retardance, mildew resistance and ultraviolet aging resistance properties.

## Figures and Tables

**Table 1:** Environmental performance of wood–plastic particleboard.

Test Item/Test Method	Test Value	Standard Value
Formaldehyde/perforator test	0.2 mg/100 g	≤9 mg/100 g
Formaldehyde/desiccator test	0.2 mg/L	≤1.5 mg/L
TVOC/72 h emission rate	0.01 mg/(m^2^·h)	≤0.5 mg/(m^2^·h)

**Table 2:** Water resistance of some thermoplastic-bonded wood-based panels [25,33,34,65].

Particleboard Type	Adhesive Dosage	24 h TS/%	IBS/MPa
Recycled PE-bonded poplar particleboard	30~70%	0.8~8.0	1.0~2.5
Recycled PP-bonded poplar particleboard	30~70%	1.9~12.0	1.1~2.5
Recycled PS-bonded poplar particleboard	30~70%	1.3~17.4	4.0~4.4
PE film-bonded poplar plywood	184 g/m^2^	5.9	Type II
PP film-bonded eucalyptus plywood	150 g/m^2^	6.8	Type I
PVC film-bonded eucalyptus plywood	320 g/m^2^	—	Type II
UF resin-bonded poplar plywood	320 g/m^2^	6.5	Type II
UF resin-bonded eucalyptus plywood	320 g/m^2^	7.5	Type II

**Table 3:** Mechanical properties of plywood bonded with different adhesives [22,30,35,36,69].

Adhesives	Hot-Pressing Temperature (°C)	Type II Bonding Strength (MPa)	Type I Bonding Strength (MPa)	MOR (MPa)	MOE (MPa)
PE film	152	1.5	0	82.8	7480
PP film	180	1.9	1.4	109.3	13,890
PVC film	183	1.1	0.5	65.1	8600
PS	140	1.25	—	79.7	5253
PHB	170	1.19	—	58.3	6001
UF resins	115~120	1.3~1.4	0	75.7~94.5	7520~13,019

## Data Availability

Not applicable.

## References

[1] Wang J., Cao X., Liu H. (2021). A Review of the Long-term Effects of Humidity on the Mechanical Properties of Wood and Wood-based Products. Eur. J. Wood Prod..

[2] Papadopoulos A. (2019). Advances in Wood Composites. Polymers.

[3] Švajlenka J., Kozlovská M. (2020). Evaluation of the Efficiency and Sustainability of Timber-based Construction. J. Clean. Prod..

[4] Liu R., Liu M., Qu Y., Huang A., Ma E. (2018). Dynamic Moisture Sorption and Formaldehyde Emission Behavior of Three Kinds of Wood-based Panels. Eur. J. Wood Prod..

[5] Treu A., Bredesen R., Bongers F. (2020). Enhanced Bonding of Acetylated Wood with an MUF-based Adhesive and a Resorcinol-formaldehyde-based Primer. Holzforschung.

[6] Tang Q., Tan H., Wei Q., Cui H., Guo W., Zhou G. (2019). Preparation of Low Formaldehyde Emission of High-density Fiberboard and Its Properties. J. For. Eng..

[7] Pizzi A., Mittal K.I. (2003). Handbook of Adhesive Technology, Revised and Expanded.

[8] Salthammer T., Mentese S., Marutzky R. (2010). Formaldehyde in the Indoor Environment. Chem. Rev..

[9] (2016). Formaldehyde Emission Standards for Composite Wood Products.

[10] Park B., Lee S., Roh J. (2009). Effects of Formaldehyde/Urea Mole Ratio and Melamine Content on the Hydrolytic Stability of Cured Urea-melamine-formaldehyde Resin. Eur. J. Wood Prod..

[11] Que Z., Furuno T., Katoh S., Nishino Y. (2007). Effects of Urea-formaldehyde Resin Mole Ratio on the Properties of Particleboard. Build. Environ..

[12] Hematabadi H., Behrooz R., Shakibi A., Arabi M. (2012). The Reduction of Indoor Air. Formaldehyde from Wood Based Composites Using Urea Treatment for Building Materials. Constr. Build. Mater..

[13] Pang H., Wang Y., Chang Z., Xia C., Han C., Liu H., Li J., Zhang S., Cai L., Huang Z. (2021). Soy Meal Adhesive with High Strength and Water Resistance Via Carboxymethylated Wood Fiber-induced Crosslinking. Cellulose.

[14] Karthuser J., Biziks V., Mai C., Militz H. (2021). Lignin and Lignin-Derived Compounds for Wood Applications—A Review. Molecules.

[15] Pizzi A. (2006). Recent Developments in Eco-efficient Bio-based Adhesives for Wood Bonding: Opportunities and Issues. J. Adhes. Sci. Technol..

[16] Yang I., Kuo M., Myers D., Pu A. (2006). Comparison of Protein-based Adhesive Resins for Wood Composites. J. Wood Sci..

[17] Sellers T. (2001). Wood Adhesive Innovations and Applications in North America. Forest Prod. J..

[18] Fang L., Chang L., Guo W., Wang Z. (2012). Overview on Adhesives and Relevant Modification for Environmentally-Friendly Plywood. China Wood Ind..

[19] Wilkowski J., Borysiuk P., Górski J., Czarniak P. (2013). Analysis of relative machinability indexes of wood particle boards bonded with waste thermoplastics. Drewno.

[20] Grinbergs U., Kajaks J., Reihmane S. (2010). Usage of Ecologically Perspective Adhesives for Wood Bonding. Sci. J. Riga Techol. Univ. Mater. Sci. Appl. Chem..

[21] Kajaks J., Reihmane S., Grinbergs U., Kalnins K. (2012). Use of innovative environmentally friendly adhesives for wood veneer bonding. Proc. Est. Acad. Sci..

[22] Kajaks J., Bakradze G., Viksne A., Reihmane S., Kalnins M., Krutohvostov R. (2009). The use of polyolefins-based hot melts for wood bonding. Mech. Compos. Mater..

[23] Han G.S. (1990). Preparation and Physical Properties of Moldable Wood-Plastic Composites. Ph.D. Thesis.

[24] Wang Z., Guo W. (2000). Environment-Friendly Plywood Production Process. CN Patent.

[25] Fang L., Chang L., Guo W., Ren Y., Wang Z. (2013). Preparation and Characterization of Wood-plastic Plywood Bonded with High Density Polyethylene Film. Eur. J. Wood Prod..

[26] Fang L., Xiong X., Wang X., Chen H., Mo X. (2017). Effects of Surface Modification Methods on Mechanical and Interfacial Properties of High-density Polyethylene-bonded Wood Veneer Composites. J. Wood. Sci..

[27] Lin T., Guo W., Gao L., Chang L., Wang Z. (2011). Effect of Cotton Stalk Pattern and Screening Size on Properties of Cotton Stalk/Recycled Plastic Composite Panels. J. For. Environ..

[28] Lin T., Guo W., Gao L., Wang Z. (2011). Effect of Particle Size on Properties of Cotton Stalk-Recycled Plastic Composites. J. Southwest For. Univ..

[29] Yu P., Zhang W., Chen M., Zhou X. (2020). Plasma-treated Thermoplastic Resin Film as Adhesive for Preparing Environmentally-friendly Plywood. J. For. Eng..

[30] Tang L., Zhang Z., Qi J., Zhao J., Feng Y. (2011). The Preparation and Application of a New Formaldehyde-free Adhesive for Plywood. Int. J. Adhes. Adhes..

[31] Qi C.S. (2013). Fabrication of Oriented Biomass-High Density Polyethylene Composites using Hot Pressing Process and Its Molding Mechanism. Ph.D. Thesis.

[32] Xia N. (2013). Fabrication and Manufacturing Mechanism of Oriented Cotton Stalk-Polypropylene Film Boards. Ph.D. Thesis.

[33] Wang D.D. (2016). A Study of the Manufacturing and Strengthening Mechanism of Composite Materials Made from Eucalyptus Veneer and Polyvinyl Chloride (PVC) Film. Ph.D. Thesis.

[34] Li X.F. (2015). Research on the Pressing Crafts and Strengthening Mechanism of Composite Materials from Eucalyptus Veneer and Polypropylene (PP) Film. Ph.D. Thesis.

[35] Demirkir C., Öztürk H., Çolakoğlu G. (2017). Effects of Press Parameters on Some Technological Properties of Polystyrene Composite Plywood. Kast. Univ. J. For. Fac..

[36] Chen Z., Wang C., Cao Y., Zhang S., Song W. (2018). Effect of Adhesive Content and Modification Method on Physical and Mechanical Properties of Eucalyptus Veneer–Poly-β-Hydroxybutyrate Film Composites. For. Prod. J..

[37] Fang L., Chang L., Guo W.Z. (2013). Analysis of Optimization Manufacturing Technology of Poplar Plywood Glued with High Density Polyethylene Film. China Wood Ind..

[38] Fang L., Zeng J., Liao X., Zou Y., Shen J. (2019). Tensile Shear Strength and Microscopic Characterization of Veneer Bonding Interface with Polyethylene Film as Wood Adhesive. Sci. Adv. Mater..

[39] Kucher M., Yakovleva O., Chyzhyk J. (2020). Thermal Expansion and Shrinkage of Unidirectional Composites at Elevated Temperatures. Strength Mater..

[40] Peng X., Zhang Z. (2018). Hot-pressing Composite Curling Deformation Characteristics of Plastic Film-reinforced Pliable Decorative Sliced Veneer. Compos. Sci. Technol..

[41] Wang H., Zhang X., Guo S., Liu T. (2021). A Review of Coextruded Wood–plastic Composite. Polym. Compos..

[42] Zhang L., Chen Z., Dong H., Fu S., Ma L., Yang X. (2021). Wood Plastic Composites Based Wood Wall’s Structure and Thermal Insulation Performance. J. Bioresour. Bioprod..

[43] Chen H., Chen T., Hsu C. (2006). Effects of wood particle size and mixing ratios of HDPE on the properties of the composites. Eur. J. Wood Prod..

[44] Ayrilmis N., Benthien J., Thoemen H., White R. (2012). Effects of fire retardants on physical, mechanical, and fire properties of flat-pressed WPCs. Eur. J. Wood Prod..

[45] Benthien J., Thoemen H. (2012). Effects of Raw Materials and Process Parameters on the Physical and Mechanical Properties of Flat Pressed WPC Panels. Compos. Part A Appl. Sci..

[46] Ayrilmis N., Jarusombuti S. (2011). Flat-pressed Wood Plastic Composite as an Alternative to Conventional Wood-based Panels. J. Compos. Mater..

[47] Friedrich D. (2021). Thermoplastic Moulding of Wood-Polymer Composites (WPC): A Review on Physical and Mechanical Behaviour under Hot-pressing Technique. Compos. Struct..

[48] Borysiuk P., Zbieć M., Jenczyk-Tołłoczko I., Jabłoński M. Thermoplastic bonded composite chipboard Part 1—Mechanical properties. Proceedings of the 8th International Science Conference: “Chip and Chipless Woodworking Processes”.

[49] Zbieć M., Borysiuk P., Mazurek A. Thermoplastic bonded composite chipboard Part 2—Machining tests. Proceedings of the 8th International Science Conference: “Chip and Chipless Woodworking Processes”.

[50] Borysiuk P., Wilkowski J., Krajewski K., Auriga R., Skomorucha A., Auriga A. (2020). Selected Properties of Flat-pressed Wood-polymer Composites for High Humidity Conditions. Bioresources.

[51] Satyanarayana K., Arizaga G., Wypych F. (2009). Biodegradable Composites Based on Lignocellulosic Fibers—An Overview. Prog. Polym. Sci..

[52] He C., Hou R., Xue J., Zhu D. (2012). Performances of PP Wood-plastic Composites with Different Processing Methods. Trans. CSAE.

[53] Schmidt H., Benthien J., Thoemen H. (2013). Processing and Flexural Properties of Surface Reinforced Flat Pressed WPC Panels. Eur. J. Wood Prod..

[54] Guo W., Wang Z. (2014). Application and Production Technology of Wood Plastic Particleboard. China Wood-Based Panels.

[55] Rahman K., Islam M., Ratul S., Dana N., Musa S., Hannan M. (2018). Properties of Flat-pressed Wood Plastic Composites as A Function of Particle Size and Mixing Ratio. J. Wood Sci..

[56] Migneault S., Koubaa A., Erchiqui F., Chaala A., Englund K., Wolcott M. (2009). Effects of Processing Method and Fiber Size on the Structure and Properties of Wood-plastic Composites. Compos. Part A Appl. S..

[57] Qi C., Guo K., Gu R., Liu Y. (2010). Preparation Technology of Oriented Cotton Stalk Bunches/High Density Polyethylene Composite Panels. Trans. CSAE.

[58] Wang Z., Guo W., Gao L. (2005). Properties, Applications and Development Trends of Wood-plastic Composite Particleboard. China Wood-Based Panels.

[59] Li Z., Wang W. (2017). Preparation and Properties of Polypropylene Based Composites with High Wood Fibers Content. J. For. Eng..

[60] Borysiuk P., Mamiński M., Parzuchowski P., Zado A. (2010). Application of Polystyrene as Binder for Veneers Bonding—The Effect of Pressing Parameters. Eur. J. Wood Prod..

[61] Tasooji M., Wan G., Lewis G., Wise H., Frazier C. (2017). Biogenic Formaldehyde: Content and Heat Generation in the Wood of Three Tree Species. ACS Sustain. Chem. Eng..

[62] (2001). Indoor Decorating and Refurbishing Materials—Limit of Formaldehyde Emission of Wood-based Panels and Finishing Products.

[63] (2010). Technical Requirement for Environmental Labeling Products Wood Based Panels and Finishing Products.

[64] Xia N., Chen X., Guo K. (2015). Optimal Film Content Improving Mechanical and Water Absorption Properties of Oriented Cotton Stalk-plastic Boards. Trans. CSAE.

[65] Wang Z., Zhao X., Guo W. (2005). Process Factors and Performances of Recycled Plastic-Wood Fiber Composites. J. Beijing For. Univ..

[66] Bekhta P., Müller M., Hunko I. (2020). Properties of Thermoplastic-Bonded Plywood: Effects of the Wood Species and Types of the Thermoplastic Films. Polymers.

[67] Sun Y., Guo L., Liu Y., Wang W., Song Y. (2019). Glue Wood Veneer to Wood-fiber-high-density-polyethylene Composite. Int. J. Adhes. Adhes..

[68] Liu Y., Li X., Wang W., Sun Y., Wang H. (2019). Decorated Wood Fiber/high Density Polyethylene Composites with Thermoplastic Film as Adhesives. Int. J. Adhes. Adhes..

[69] Shen T., Sun Y., Liu Y., Wang W., Shan W. (2020). Study on Preparation and Adhesion Property of Veneer Overlaid Wood Flour/high Density Polyethylene Composites. J. For. Eng..

[70] Chang L., Tang Q., Gao L., Fang L., Wang Z., Guo W. (2016). Fabrication and Characterization of HDPE Resins as Adhesives in Plywood. Eur. J. Wood Prod..

[71] Kamke F., Lee J. (2007). Adhesive Penetration in Wood—A review. Wood Fiber Sci..

[72] Luedtke J., Gaugler M., Grigsby W., Krause A. (2019). Understanding the Development of Interfacial Bonding within PLA/wood-based Thermoplastic Sandwich Composites. Ind. Crop. Prod..

[73] Fang L., Yin Y., Han Y., Chang L., Wu Z. (2016). Effects of Number of Film Layers on Properties of Thermoplastic Bonded Plywood. J. For. Eng..

[74] Bekhta P., Sedliačik J. (2019). Environmentally-Friendly High-Density Polyethylene-Bonded Plywood Panels. Polymers.

[75] Li X., Ren C., Wei W., Zhang S. (2015). Pressing Crafts and Mechanical Properties of Eucalyptus Veneer/Polypropylene (PP) Film Composites. J. Northeast For. Univ..

[76] Xia N., Chen Q., Guo K. (2015). Fabrication Technology Optimization of Oriented Cotton Stalk-polypropylene Film Boards. Trans. CSAE.

[77] Gao Y.L. (2018). Process Optimization of Plywood Made of PVC Film and Eucalyptus Veneer. Master’s Thesis.

[78] Wu Y. (2020). Process Optimization of PVC Film and Eucalyptus Veneer to Prepare Composite Plywood. Int. Wood Ind..

[79] Tian F., Chen L., Xu X. (2021). Dynamical Mechanical Properties of Wood-High Density Polyethylene Composites Filled with Recycled Rubber. J. Bioresour. Bioprod..

[80] Peng X., Zhang Z., Zhao L. (2020). Analysis of Raman Spectroscopy and XPS of Plasma Modified Polypropylene Decorative Film. J. For. Eng..

[81] George J., Sreekala M., Thomas S. (2001). A Review on Interface Modification and Characterization of Natural Fiber Reinforced Plastic Composites. Polym. Eng. Sci..

[82] Mohit H., Arul Mozhi Selvan V. (2018). A comprehensive review on surface modifcation, structure interface and bonding mechanism of plant cellulose fiber reinforced polymer based composites. Compos. Interfaces.

[83] Xia N., Li F., Liu K., Shao Y., Xing S., Guo K.Q. (2020). Effects of Coupling Agents on the Properties of Cotton Stalk-polypropylene Film Boards. Bioresources.

[84] Wei S., Zhang S., Fei B., Zhao R. (2021). Effect of Monomer Type on Polydopamine Modification of Bamboo Flour and the Resulting Interfacial Properties of Bamboo Plastic Composites. Ind. Crop. Prod..

[85] Zhou X., Cao Y., Yang K., Yu P., Chen W., Wang S., Chen M. (2020). Clean Plasma Modification for Recycling waste Plastic Bags: From Improving Interfacial Adhesion with Wood Towards Fabricating Formaldehyde-free Plywood. J. Clean. Prod..

[86] Ye C., Yang W., Xu J., Chen Z., Liao R., Zhong Z. (2015). Hot-pressing Technology of Formaldehyde-free Plywood Made by Veneers with Rolling Holes and High-density Polyethylene Film. J. Northwest A&F Univ. (Nat. Sci. Ed.).

[87] Li Z., Qi X., Gao Y., Zhou Y., Chen N., Zeng Q., Fan M., Rao J. (2019). Effect of PVC Film Pretreatment on Performance and Lamination of Wood-plastic Composite Plywood. RSC Adv..

[88] Gu L., Ding T., Jiang N. (2019). Development of Wood Heat Treatment Research and Industrialization. J. For. Eng..

[89] Ayrilmis N., Jarusombuti S., Fueangvivat V., Bauchongko P. (2011). Effect of Thermal-Treatment of Wood Fibres on Properties of Flat-pressed Wood Plastic Composites. Polym. Degrad. Stabil..

[90] Follrich J., Muller U., Gindl W. (2006). Effects of Thermal Modification on the Adhesion between Spruce Wood and a Thermoplastic Polymer. Eur. J. Wood Prod..

[91] Fang L., Chang L., Guo W., Chen Y., Wang Z. (2014). Effect of High Temperature on Properties of Wood/Plastic Plywood. China Wood Ind..

